# A Qualitative Research Study Comparing the Quality of Life of Implant Treated and Endodontically Treated Patients

**DOI:** 10.3290/j.ohpd.c_2283

**Published:** 2025-09-26

**Authors:** Gülçin Cagay Sevencan, Hilal Gülgezen Aydin

**Affiliations:** a Gülçin Cagay Sevencan Department of Endodontics, Faculty of Dentistry, Tekirdağ Namık Kemal University, Tekirdag, Turkey. Idea concept, hypothesis and performed the experiments in partial fulfilment of requirements for a degree, wrote and proofread the manuscript.; b Hilal Gülgezen Aydin Department of Prosthodontics, Faculty of Dentistry, Tekirdağ Namık Kemal University, Tekirdag, Turkey. Performed the experiments in partial fulfilment of requirements for a degree, wrote and proofread the manuscript.

**Keywords:** dental implants, oral health impact profile, quality of life, endodontics, oral health

## Abstract

**Purpose:**

In recent years, advancements in technology have led to significant developments in the fields of dental implants and endodontic treatment. These technological improvements have resulted in better treatment outcomes and enhanced quality of life for patients undergoing these procedures. The objective of this study is to compare the quality of life related to oral health from the patients’ perspective between single-tooth root canal treatment (RCT) and single-tooth dental implant (DI) treatment.

**Methods:**

Patients in the RCT (n = 21) and DI (n = 19) groups, treated by experienced endodontists and prosthodontists, completed a two-part questionnaire after permanent restorations. The first part collected demographic and socioeconomic data, while the second part included a shortened version of the Oral Health Impact Profile (OHIP-14). Six focus group discussions were conducted for thematic analysis. For the comparison of OHIP-14 sub-dimension and total scores by groups, the independent samples t-test and the Mann-Whitney U test were used, while the Fisher Freeman-Halton test and Yates’ continuity correction evaluated categorical data (α = 0.05).

**Results:**

Statistically significant differences favoured DI in total OHIP scores (P = 0.041), social disability (P = 0.028), and psychological disability (P = 0.012). No statistically significant differences in mean total OHIP values were found between socioeconomic classes for either treatment (RCT; P = 0.892, DI; P = 0.572).

**Conclusion:**

The results of this study indicate that the overall OHIP scores were comparable, with a high level of satisfaction observed for both treatment modalities. Content analysis of the discussion groups revealed several themes. These can be listed as: importance of preserving the tooth; dentist visits; treatment costs; physical pain; psychological discomfort; aesthetics; function; and treatment satisfaction. However, patients noted that both treatments improved their oral health and they valued the preservation of natural teeth.

The primary objective of dental implant and endodontic treatments is to provide patients with options that yield the most favourable long-term outcomes. In clinical practice, the decision-making process regarding whether to pursue endodontic treatment or dental implant treatment for painful teeth with material loss is influenced by a multitude of factors.^9,23^ The patient’s choice of treatment, as recommended by the clinician, is affected by various factors, including the patient’s psychological state and levels of anxiety, the intensity of pain, oral hygiene practices and dental care habits, the patient’s overall health status and any existing medical conditions, the clinical condition of the affected tooth and the necessity for restoration, the prognosis of the available treatment options, the anticipated outcomes of the treatments, the extent of coverage provided by health insurance, the overall cost of treatment, and the expected duration of the treatment process.^18,21^

Recent advancements in technology related to dental implants and endodontic procedures have substantially elevated patient expectations. These improvements in treatment processes and outcomes have a positive impact on patients’ quality of life.

The success and survival rates of root canal treatment (RCT) and dental implants (DI) have been extensively documented in the literature.^8,13^ However, clinical parameters alone are inadequate for assessing oral health outcomes.^3,8,15^ A paradigm shift has occurred in oral healthcare, emphasising patient-centred outcomes for treatments and services. This shift is also evident in the broader context of healthcare.^1,11^ Oral health-related quality of life indices complement clinical measurements, as health encompasses not only the absence of disease but also the presence of psychological, physical, and social well-being.^7^

The evaluation of the psychosocial impact of health conditions on patients’ quality of life and their perceptions is of paramount importance and should be considered by healthcare professionals.^22^ The Oral Health Impact Profile (OHIP-14) is a validated instrument designed to provide a multidimensional assessment of the disability resulting from oral health issues.^20^ Qualitative research contributes to a deeper understanding of specific concerns.^2^ Focus group discussions facilitate the exchange of ideas, insights, and experiences within a social context, enabling individuals with similar backgrounds and experiences to support one another and reflect on their perspectives in relation to those of others.^14^

Numerous studies have evaluated the oral health-related quality of life associated with RCT and DI; however, there is a paucity of research directly comparing these two treatment modalities from the patient’s perspective.^6^ In recent years, advancements in technology related to both dental implants and endodontic treatments have aligned with patient expectations, resulting in significant changes to the treatment process and outcomes, thereby enhancing patients’ quality of life.

This study aims to evaluate the evolution of therapeutic interventions in dentistry and their effects on patients’ quality of life. The primary objective of this study is to compare the oral health-related quality of life associated with two treatment options: single-tooth root canal treatment and single dental implant. Both treatment modalities have demonstrated improved success and survival rates, which can be attributed to advancements in technology, as perceived by patients. Additionally, the study will include demographic data, such as socioeconomic status, oral and dental health habits, and overall health status, as these factors may significantly influence patients’ perceptions and treatment decisions.

The null hypothesis of this study posits that there is no significant difference between endodontic treatment and dental implant treatment in terms of total OHIP-14 scores, which serve as indicators of patients’ quality of life.

## MATERIALS AND METHODS

### Subject Recruitment and Inclusion Criteria

The sample size was determined through power analysis (G*Power v3.1). Fueki et al reported a reduction in the OHIP summary score from 29.9 at baseline to 14.7 and 20.2 at 6 and 12 months after IFPD treatment, respectively,^5^ indicating an effect size ranging from 0.4 to 0.6. Consequently, a moderate effect size (d Z 0.5) was established based on the criteria proposed by Cohen.^14^ The required sample size was calculated to be 34, which would provide 80% power at a 5% significance level. To account for potential participant dropout, the final sample size was adjusted to 42. The study design adhered to the principles outlined in the STROBE statement (https://www.strobe-statement.org/checklists/).

Ethics committee approval was obtained from the Non-Interventional Ethics Committee of Tekirdag Namik Kemal University (protocol number: 2022.168.09.15). This study evaluated post-doctoral endodontic and prosthodontic clinics where patients received treatment. A random selection was conducted from a database comprising the files of treated patients. To ensure an adequate duration had elapsed following the occlusal rehabilitation of patients who underwent single-tooth implants and single-tooth root canal treatments, a minimum interval of 6 months was established. A total of 42 patients from both treatment groups were contacted via telephone to participate in the study. Ethical approval was secured from the relevant ethics committee, and all study materials and methodologies were approved prior to the commencement of the research. Patients who chose to participate were informed that they would be invited to engage in focus group discussions, during which they would respond to survey questions and participate in in-person dialogues with their respective dentists.

The inclusion criteria for this study were as follows: (1) patients with a single-tooth dental implant (DI) or a single-tooth root canal treatment (RCT); (2) patients who have maintained occlusal function for a minimum of 6 months; (3) clinicians with equivalent levels of expertise (specialists in each clinic); (4) patients aged 18 years or older, and (5) patients classified as American Society of Anesthesiologists (ASA) class I or II.

Patients in the group undergoing dental implant treatment were selected from individuals who had received implants at least 2 months following tooth extraction, with restorations completed using a late loading protocol. In contrast, patients in the endodontic treatment group were selected based on the presence of vital pulp, which required at least a two-surface restoration, achieved through direct restoration methods. Two patients from the implant treatment group, who had initially consented to participate in the study, were subsequently excluded due to their failure to attend scheduled follow-up appointments. Consequently, the study proceeded with a total of 21 participants in the endodontic treatment group and 19 participants in the implant treatment group.

### Collection of Demographic and Socioeconomic Data

In the initial phase of the study, participants completed a questionnaire designed to collect demographic and socioeconomic information. A modified Kuppuswamy socioeconomic scale was utilised to assess socioeconomic status.^12,16^ The original scale, which categorizes socioeconomic status into five distinct levels, was adapted for statistical analysis based on the number of participants in the study, resulting in a classification into three socioeconomic levels: upper, middle, and lower socioeconomic groups. The health status of the participants was evaluated by measuring blood pressure and pulse rate. Additionally, weight and height were recorded to calculate body mass index (BMI). Participants were also asked about their oral hygiene practices and smoking habits.

### Quality of Life Assessment: Oral Health Impact Profile

In the second section of the questionnaire, participants were instructed to complete the quality-of-life assessment, which is an abbreviated version of the OHIP-14, immediately prior to the focus group interview.^19^ The primary aim of this index is to provide a comprehensive measurement of self-reported dysfunction, discomfort, and disability attributable to oral diseases. This instrument is specifically designed to evaluate individuals’ perceptions regarding the social implications of oral diseases on their overall well-being.^20^ The adaptation proposed seven dimensions illustrating the impact of oral health on patients’ well-being. The instrument comprises 49 items, with seven items corresponding to each dimension. These dimensions include functional limitation, psychological discomfort, physical pain, physical disability, social disability, psychological disability, and handicap. A condensed version, referred to as the OHIP-14, was subsequently developed through controlled stepwise regression analyses, resulting in a subset of 14 items.^19^ Each item is scored on a five-point scale, with responses ranging from ‘never’ (coded as 0) to ‘very often’ (coded as 4).

### Focus Group Discussions

A semi-structured discussion guide, previously utilised in an earlier study, was employed by the moderator dentist during the group sessions.^6^ The guide included questions related to oral health-related quality of life, as well as general perceptions of oral health, dental experiences, and treatment experiences (Table 1).^6^ Two specialist dentists participated in both discussion groups. The discussions were either transcribed in writing or audio-recorded using a digital recorder by the moderator for subsequent data analysis. Each discussion lasted 90 minutes during which the needs of all participants were addressed.

**Table 1:** Discussion guide questions


1. Before you received your endodontic treatment or implant, how did you feel about the importance of keeping your own teeth?
2. How often did you visit the dentist before your treatment? What was your main reason for visiting the dentist?
3. After your treatment, how often did you visit the dentist? What was your main reason for visiting the dentist?
4. Describe your daily life experience since your treatment.
5. How does your endodontically treated tooth or implant feel compared to your other teeth?
6. How does your endodontically treated tooth or implant affect your ability to eat? Drinking? Does it feel different to eat or drink now?
7. How does your endodontically treated tooth or implant affect your appearance? How has it affected your appearance and smile?
8. Thinking back to the procedure when you had your endodontic treatment or implant, how would you rate the pain? What was your level of pain after the procedure? Currently?
9. Can you describe any problems or concerns with maintaining your implant or endodontically treated tooth?
10. If you had to return for maintenance, how many times and what type of procedures have you had?
11. Are you satisfied with the outcome of your root canal-treated tooth or implant? *
*The questions are referenced from Gatten et al.^20^

### Data Analysis

The results were analysed using SPSS Version 23 (IBM). According to the Shapiro–Wilk test, only the Psychological Discomfort dimension showed a normal distribution within the groups, while the other dimensions did not exhibit a normal distribution. Since the number of cases in the groups is less than 50, normality was assessed using the Shapiro–Wilk test. For the comparison of OHIP-14 sub-dimension and total scores between groups, the independent samples t-test was used if the data were normally distributed, and the Mann–Whitney U test was used if the data were not normally distributed. For the independent samples t-test, the homogeneity of variances between the two groups was examined using Levene’s test. Fisher’s Freeman-Halton test and Yates’ continuity correction were used to evaluate categorical data. A significance level of P <0.05 was established.

The analysis of the focus group discussions was conducted using transcripts obtained from digital recordings. These recordings were either transcribed manually or captured directly. The resulting data were analysed for content and subsequently reviewed for accuracy. The evaluation of the focus group outcomes was performed by two individuals who collaborated to establish a thematic coding scheme. In instances of disagreement regarding themes and sub-themes, discussions were facilitated to achieve a consensus.

## RESULTS

A total of 40 individuals participated in six focus interviews. Participants were categorised into two treatment groups and further divided into three subgroups based on the number of sessions attended. The DI group (n = 19) consisted of 13 males and 6 females, while the RCT group (n = 21) included 9 males and 12 females. The mean age of the participants was 44.2 years. Among those who received implant treatment, 5 individuals were treated for anterior tooth loss, and 14 were treated for posterior tooth loss. In the RCT group, 6 participants received treatment for anterior teeth, while 15 were treated for posterior teeth. A statistically significant correlation was identified between the socioeconomic status of the patient group receiving endodontic treatment and that of the patient group receiving implant treatment, with the implant treatment group exhibiting a higher socioeconomic status (P = 0.019). No statistically significant differences were observed between the groups concerning other demographic variables. An examination of the categorical data by group is presented in Table 2.

**Table 2:** Analysis of categorical data by groups

	RCT	DI	Total	Statics	P
Socioeconomic status
Upper	8 (38.1)	14 (73.7)	22 (55)	–	0.025^x^
Middle	6 (28.6)	0 (0)	6 (15)		
Lower	7 (33.3)	5 (26.3)	12 (30)		
Gender
Female	12 (57.1)	6 (31.6)	18 (45)	1.702	0.192^y^
Male	9 (42.9)	13 (68.4)	22 (55)		
Marital status					
Married	13 (61.9)	12 (63.2)	25 (62.5)	–	>0.999^x^
Single	5 (23.8)	4 (21.1)	9 (22.5)		
Divorced	3 (14.3)	3 (15.8)	6 (15)		
Toothbrushing
Never	1 (4.8)	0 (0)	1 (2.5)	–	0.553^x^
Rarely	4 (19.1)	2 (10.5)	6 (15)		
Once a day	3 (14.3)	3 (15.8)	6 (15)		
Twice a day or more	13 (61.9)	14 (73.7)	27 (67.5)		
Dental flossing
Never	10 (47.6)	9 (47.4)	19 (47.5)	–	0.312 ^x^
Rarely	4 (19.1)	6 (31.6)	10 (25)		
Once a day	5 (23.8)	1 (5.3)	6 (15)		
Twice a day or more	2 (9.5)	3 (15.8)	5 (12.5)		
Mouthwash
Yes	9 (42.9)	9 (47.4)	18 (45)		
No	12 (57.1)	10 (52.6)	22 (55)	0.000	>0.999 ^y^
Treatment region
Anterior	6 (28.6)	5 (26.3)	11 (27.5)	0.000	>0.999^y^
Posterior	15 (71.4)	14 (73.7)	29 (72.5)		
Smoking habit
Yes	6 (28.6)	6 (31.6)	12 (30)	0.043	0.836^x^
No	15 (71.4)	13 (68.4)	28 (70)		
Body mass index
Underweight	4 (19.1)	0 (0)	4 (10)	–	0.254 ^x^
Normal weight	10 (47.6)	10 (52.6)	20 (50)		
Overweight	5 (23.8)	7 (36.8)	12 (30)		
Obesity	2 (9.5)	2 (10.5)	4 (10)		
^y^ Yates’ continuity correction; ^x^ Fisher Freeman–Halton; n (%)

**Fig 1:**
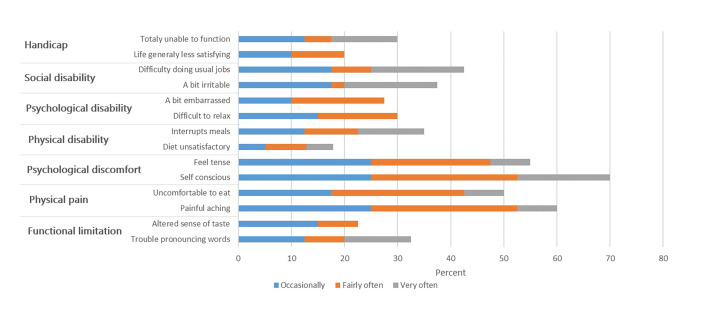
The percentage distribution of questions across the seven dimensions for ‘sometimes’ to ‘very often,’ excluding ‘never’ and ‘rarely’ categories.

Upon conducting a comprehensive review of all OHIP items, it was found that a significant majority of participants (30% to 82%) indicated that they had never experienced any adverse effects on their oral health-related quality of life following treatment. Figure 1 presents the distribution of responses across the seven dimensions, categorised from ‘sometimes’ to ‘very often’. It is important to note that the total percentage of responses does not equal 100% as it excludes the categories ‘never’ and ‘rarely’. The most reported impacts on oral health-related quality of life among participants included physical pain, with 60% reporting aching pain and 50% experiencing discomfort while eating, as well as psychological discomfort, with 70% indicating feelings of self-consciousness and 55% reporting feelings of nervousness.

The treatment groups demonstrated statistically significant differences across the OHIP items. The DI group recorded a mean OHIP-14 score of 4, whereas the RCT group exhibited a mean score of 9. A statistically significant difference in the mean severity score was identified between the two treatment categories (U = 275, P = 0.041). Further analysis of the mean severity score across each of the seven dimensions revealed statistically significant differences between the treatment groups in two of the seven dimensions: social disability (U = 273, P = 0.028) and psychological disability (U = 281.5, P = 0.012) (Table 3). A higher score on the OHIP-14 indicates a poorer oral health-related quality of life.

**Table 3:** Comparison of OHIP-14 sub-dimension and total scores by groups

	RCT	DI	Total	Statics	p
Functional limitation	0 (0–1)	0 (0–1)	0 (0–1)	U = 201.500	0.962^x^
Physical pain	2 (0.5–3)	1 (0–3)	2 (0–3)	U = 251.000	0.158 ^x^
Psychological discomfort	3 ± 1.9	2.1 ± 1.5	2.6 ± 1.8	t = 1.706	0.096^z^
Physical disability	0 (0–2)	0 (0–2)	0 (0–1)	U = 251.000	0.079^x^
Social disability	1 (0–3)	0 (0–1)	0 (0–2)	U = 273.000	**0.028** ^x^
Psychological disability	1 (0–2)	0 (0–1)	0 (0–1)	U = 281.500	**0.012** ^x^
Handicap	0 (0 – 0.5)	0 (0–1)	0 (0–1)	U = 192.000	0.809^x^
OHIP Total	9 (6.5–14)	4 (2–11)	7 (3–11.8)	U = 275.000	**0.041** ^x^
^x^ Mann–Whitney U Test (U); ^z^ Independent t-test (t); median (Q1–Q3); mean ± SD.

Upon further analysis of socioeconomic status within both treatment groups, no statistically significant differences were observed in the mean OHIP total values across socioeconomic classes in the RCT group (P = 0.892). The mean OHIP total values were recorded as 9.8 for the upper class, 9.5 for the middle class, and 11.3 for the lower class. In the DI group, the median OHIP total values did not exhibit statistically significant variation based on socioeconomic class (U = 29,5, P = 0.572). Notably, there were no participants classified within the middle socioeconomic class. The median OHIP total value was 3.5 for the upper socioeconomic group and 6 for the lower group, with no statistically significant difference between these two groups (U = 27, P = 0.484). Additionally, factors such as gender, marital status, frequency of oral hygiene practices, smoking habits, and BMI values did not show statistically significant differences between the groups.

A content analysis of the discussion groups was performed, which identified the statements most frequently articulated by the participants. The resulting data were subsequently categorised into themes (Table 4). The predominant themes encompassed the significance of preserving natural teeth, the necessity of regular dental visits, perceptions regarding treatment outcomes, aesthetic considerations, functionality, and overall satisfaction with the treatment received.

**Table 4:** Themes and findings from face-to-face interviews

Themes	Results
**Importance of preserving tooth**	• Participants (RCT: 80.9%; DI: 89.4%) emphasised the importance of tooth preservation both before and after treatment; however, they expressed regret over not having provided adequate care, acknowledged a fear of tooth loss, and became aware of their need for improved care.
**Dentist visits**	• Only a small proportion of participants reported routine dental visits prior to treatment (RCT: 19%; DI: 10.5%). • Most patients tend to seek dental care only when experiencing noticeable problems or pain.
**Treatment costs**	• Patients tend to prefer retaining their natural teeth over opting for implants, primarily due to the high cost of implant treatment. • The presence of insurance coverage for root canal treatment (RCT) statistically significantly influences this decision.• Consequently, RCT is often considered before proceeding with dental implants (DI).
**Physical pain**	**Dental implant (DI) and Postoperative period** • Preoperative tooth pain is alleviated following the procedure.• Analgesics are required for a few days post-surgery.• The postoperative recovery period is generally brief. **Root canal treatment (RCT) and postoperative period** • Complete resolution of pain is achieved.• No medication is needed; only mild sensitivity is experienced for two days post-treatment.
**Psychological discomfort**	• Patients who undergo dental implant (DI) treatment tend to be cautious about protecting the implant and often express concerns regarding potential fracture or discoloration. • Patients who receive root canal treatment (RCT) report satisfaction with the preservation of their natural teeth.• RCT recipients generally prefer endodontic therapy over more invasive surgical interventions such as extractions or implants. • A subset of patients express anxiety about the possibility of recurrent pain following RCT.• The psychological impact of treatment should be carefully evaluated prior to initiating any dental intervention.
**Esthetics**	• Patients who underwent dental implant (DI) treatment reported increased self-confidence and perceived positive aesthetic improvements. • Patients who received root canal treatment (RCT) noted enhanced aesthetics following the replacement of discolored or amalgam restorations. • While patients adapted quickly to the appearance of endodontically treated teeth, adjustment to the aesthetics of implant-supported restorations required more time. • The intraoral position of the implant or natural tooth statistically significantly influenced overall patient satisfaction.
**Function**	• The RCT group reported experiencing pain upon pressure application while eating with the affected tooth; however, post-treatment, they achieved comfortable eating and drinking. • The DI group rarely encountered loosening or detachment of implant components and expressed satisfaction due to the restored function in the edentulous area. • Overall, patients reported comfort in eating and drinking comparable to that with their natural teeth.
**Treatment satisfaction**	• The majority of participants expressed satisfaction with the treatment outcomes, perceiving the restored teeth as comparable to their natural dentition. • The DI group reported minor issues such as prosthesis dislodgement and food impaction.• The RCT group experienced mild postoperative pain following treatment.


## DISCUSSION

The objective of this study was to qualitatively examine the changes in quality of life, patient expectations, and satisfaction levels among individuals undergoing single-tooth implant treatment and single-tooth endodontic treatment. The findings of this study present qualitative data indicating that patients express a high level of satisfaction with both treatment options. It is essential to acknowledge that implant therapy and endodontic treatment constitute distinct treatment modalities, each possessing its own specific indications and contraindications. Therefore, these two treatment modalities should not be viewed as direct competitors.

A recent study indicated that the majority of participants’ responses were categorised as psychological discomfort and physical pain. Patients undergoing implant treatment expressed apprehensions regarding the potential complications associated with the fracture or loss of the implant. The null hypothesis of this study has been rejected. The findings revealed that psychological discomfort was the most frequently reported complaint, followed by physical pain. The primary factor contributing to psychological discomfort was identified as a lack of self-confidence concerning dental issues.

Gatten et al. reported a statistically significant disparity in the types of treatments concerning psychological discomfort and psychological disability, as indicated by the survey results.^6^ In alignment with these findings, the present study demonstrated that elevated scores for psychological discomfort and social disability were associated with RCT. Although no statistically significant differences were observed between the treatment groups, the most frequently reported effect was physical pain, with 60% of patients experiencing aching pain and 50% reporting discomfort while eating. Psychological discomfort was also prevalent, with 70% of patients feeling self-conscious and 55% feeling nervous. A study conducted by Sanz et al. found that the only statistically significant difference pertained to physical pain; however, patients expressed high satisfaction with both treatment modalities. Specifically, ‘pain during treatment’ was perceived as statistically significant worse for RCT compared to DI.^17^ Various factors can influence the management of pain in endodontics. Patients often seek endodontic treatment while experiencing pain, whereas implants are typically planned when the patient is in a state of good health. This distinction may affect pain perceptions. Despite many patients reporting physical pain, no statistically significant differences were identified between the treatments. Patients indicated that severe pain prior to endodontic treatment nearly resolved afterwards, with only mild discomfort reported during tooth extraction prior to implant surgery.

The procedures were conducted by professionals possessing more than 10 years of experience in their respective disciplines. The results indicated that patients expressed high levels of satisfaction with both RCT and DI. A separate study assessing oral health-related quality of life (OHRQoL) and patient satisfaction following root canal treatment revealed that the highest satisfaction levels were reported among patients treated by endodontic specialists, in contrast to those treated by dental students and postgraduate trainees.^17^ This finding highlights the potential influence of the specialities of the treating professionals on the observed outcomes.

In a study evaluating OHRQoL subsequent to RCT and DI, it was found that 73.1% of patients who underwent RCT and 100% of patients who received DI reported never or almost never experiencing any of the evaluated assessment items.^6^ Similarly, within the context of this study, when all items from the OHIP were taken into account, a statistically significant majority (30–82%) of participants indicated that they had not experienced any impairment in oral health-related quality of life following treatment.

The sole complaint documented was food impaction, which was reported in 26.9% of cases involving DI and in RCT. This issue was not addressed in the OHIP-14 questionnaires and, consequently, was excluded from this study.^4^ Although ‘food impaction’ was not part of the questionnaire, patients articulated this concern during face-to-face interviews. It is essential to acknowledge that patient apprehensions regarding food retention are more closely associated with coronal restoration than with the RCT or DI procedures themselves.

Statistically significant improvements in Oral Health-Related Quality of Life (OHRQoL) and the Oral Health Impact Profile (OHIP) have been documented following both RCT and DI.^4,10^ One study reported an enhancement in OHRQoL 1 year after dental implants compared to the condition of tooth loss, while another study reported improved OHIP scores at 1 and 6 months following endodontic treatment.^4,10^ This particular study included patients who had undergone treatment at least 6 months prior, whereas Gatten et al. mandated a 1-year follow-up, and Sanz et al. conducted evaluations after a 2-year period.^6,17^ Although extended follow-up durations may enhance prognostic accuracy, retrospective studies are often constrained by the potential for patients to forget their treatment experiences. In the present study, patients reported low OHIP scores for both RCT and DI, which is consistent with findings from other research. While a difference was noted between the two treatment modalities, this distinction was not conclusive, as subjective factors may have influenced the results; nonetheless, all patients expressed high satisfaction with the treatment outcomes.^4,6^ The low total OHIP scores reported by patients for both treatments align with previous research findings.^6,17^ Additionally, patients voiced financial concerns regarding treatment options during oral interviews. A statistically significant disparity was observed in the socioeconomic distribution of patients receiving different treatments. Specifically, RCT exhibited a more equitable distribution across socioeconomic groups, whereas patients undergoing DI were predominantly categorised into high and low socioeconomic groups, with no participants identified within the middle-class socioeconomic group. This discrepancy may be attributed to the fact that RCT is typically covered by insurance, in contrast to DI. The frequency of regular dental check-ups was notably low, with only 10.5% of DI patients and 19% of RCT patients reporting attendance. However, a substantial majority – 89.4% of DI patients and 80.9% of RCT patients – acknowledged the importance of preserving natural teeth. Although no statistically significant correlation was identified between tooth position and treatment groups, interviews suggested that the positioning of teeth – anterior or posterior – affects aesthetic perceptions, with a greater emphasis placed on the preservation of anterior teeth. Yu et al. reported that the loss of anterior teeth adversely impacted patients’ oral health-related quality of life.^24^

While a medium effect size was identified in this study, conducting future research with a larger sample size is likely to yield more accurate results, as the current findings may be influenced by subjective factors. Given the qualitative nature of the study, it is essential to re-evaluate the findings across diverse populations, considering the potential impact of social differences. Furthermore, the study exclusively focused on the treatment process, lacking a comparative analysis of outcomes before and after treatment concerning tooth loss or associated anxiety, which warrants consideration. The data presented offer valuable insights for clinicians in evaluating treatment prognosis and benefits, as well as their effects on patients’ quality of life. To improve decision-making between root canal treatment and dental implants, long-term studies that compare pre- and post-treatment outcomes are necessary.

## CONCLUSION

Within the limitations of this retrospective study, overall satisfaction levels were found to be high for both dental implant and root canal treatments. Advances in clinical and experimental medicine, particularly in the fields of dental implants and endodontic treatment, have enabled the fulfillment of patient expectations in clinical settings. Consequently, the treatment processes and outcomes have positively influenced patients’ quality of life. Participants reported that both treatment options enhanced their oral health and acknowledged the importance of preserving natural teeth. Therefore, clinicians should consider the changes in patients’ quality of life and their expectations when making treatment decisions.
